# Anesthesia with sevoflurane or isoflurane induces severe hypoglycemia in neonatal mice

**DOI:** 10.1371/journal.pone.0231090

**Published:** 2020-04-02

**Authors:** Qian Yu, Jian Li, Chun-ling Dai, Hengchang Li, Khalid Iqbal, Fei Liu, Cheng-Xin Gong

**Affiliations:** 1 Department of Neurochemistry, Inge Grundke-Iqbal Research Floor, New York State Institute for Basic Research in Developmental Disabilities, Staten Island, New York, United States of America; 2 Department of Orthopedic, Shandong University Qianfoshan Hospital, Jinan, Shandong, China; 3 Department of Pediatrics, Second Xiangya Hospital, Central South University, Changsha, Hunan, China; 4 Department of Anesthesiology, Guangzhou First People’s Hospital, Guangzhou Medical University, Guangzhou, Guangdong, China; Massachusetts General Hospital, UNITED STATES

## Abstract

Sevoflurane and isoflurane are among the most commonly used general anesthetics for children including infants, but their impact on metabolism, especially on blood glucose level, in children is not well understood. We investigated the impacts of anesthesia of neonatal (7–8 days old) and adult (2–3 months old) mice with the inhalational anesthetics 2.5% sevoflurane or 1.5% isoflurane, or the injectable anesthetics propofol (150 mg/kg) or avertin (375 mg/kg), for up to 6 hours. We found that sevoflurane and isoflurane induced severe hypoglycemia in neonatal mice and that this phenomenon was specific to the inhalational anesthetics because the injectable anesthetics propofol and avertin did not induce hypoglycemia. Surprisingly, the inhalational anesthesia induced hyperglycemia instead in adult mice. We also demonstrated that the inhalational anesthesia-induced hypoglycemia was a major cause of death for the neonatal mice receiving intranasal administration of saline prior to anesthesia. These studies revealed severe hypoglycemia in neonatal mice during anesthesia with sevoflurane or isoflurane. If this phenomenon also occurs in human, our findings would warrant closely monitoring blood glucose level and maintaining it in the normal range in infants receiving inhalational anesthesia.

## Introduction

General anesthesia is essential for many surgical procedures. Maintaining vital signs and major blood biochemicals within the normal ranges during anesthesia is critical to the success of surgery’s outcome and even to the survival of patients. Among many blood biochemicals, blood glucose level was reported to increase both in humans [1] and in laboratory animals [2] during anesthesia. This increase of blood glucose level appears to represent a response to anesthesia-induced stress [3, 4] and anesthesia-induced hepatic insulin resistance [4]. Because the elevation of blood glucose induced by anesthesia is only mild to moderate, blood glucose level is not routinely monitored during anesthesia/surgery unless there are other indications such as diabetes.

Children, especially infants, are more vulnerable to insults induced by anesthesia. However, little is known about the effect of anesthesia on blood glucose level in young children or in newborn laboratory animals. One previous study reported that anesthesia of neonatal mice on postnatal day 7 (P7) with isoflurane for six hours resulted in reversible hypoglycemia [5], but this finding appears to be overlooked in the field, probably because the study aimed to investigate the effects of neonatal isoflurane exposure on brain cell viability and long-term spatial learning and memory. Blood glucose level is not routinely monitored during anesthesia/surgery for young children in clinical practice at present, unless they are born prematurely or have other high-risk conditions. Here, we report severe hypoglycemia in neonatal mice during anesthesia with sevoflurane or isoflurane, the most commonly used general anesthetics for children including infants.

## Materials and methods

### Materials and reagents

Sevoflurane and isoflurane were purchased from Henry Schein, Inc. (Melville, NY, USA). Propofol was purchased from MP Biomedicals (Solon, OH, USA). Other chemicals were from Sigma-Aldrich (St. Louis, MO, USA).

### Animals and anesthesia

The breeding pairs of mice (a hybrid of 129/Sv and C57BL/6 mice) were initially obtained from Jackson Laboratory (New Harbor, ME, USA). The mice were bred in our air-conditioned animal facility and housed with a 12/12 hr light/dark cycle and with ad libitum access to food and water. The housing, breeding, and animal experiments were approved by the Institutional Animal Care and Use Committee of the New York State Institute for Basic Research in Developmental Disabilities and were in accordance with the PHS Policy on Humane Care and Use of Laboratory Animals (revised March 15, 2010).

Mouse pups at the age of P7-8 were anesthetized in an anesthesia chamber filled with 2.5% sevoflurane or 1.5% isoflurane in a mixture of O_2_ and N_2_ (50%/50%) at a flow rate of 0.9−1 L/min for up to 6 hrs. A small Petri dish of water was placed into the anesthesia chamber for maintaining moisture. At the end of anesthesia, the sevoflurane or isoflurane was turned off, and the mouse pups were kept in the same chamber with O_2_ and N_2_ for 1 hr to allow their recovery from anesthesia. A warm pad was placed in the anesthesia chamber to maintain the body temperature of the neonatal mice to 35–36 ^o^C during the procedure. After awakening from anesthesia, the mouse pups were returned to their parents’ cages. The blood glucose level of the mice was measured at various time points by using tail blood samples (2–3 μl). The blood glucose level was measured by using a glucometer (Bayer Contour, Tarrytown, NY). For blood sampling, approximately 1-mm tip of the mouse tail was cut by using a pair of clean surgical scissors to allow bleeding for 2–3 μl blood directly onto a glucometer test strip. The bleeding was then stopped by dabbing the tail tip with a clean tissue paper. For time-course studies, blood samples (2–3 μl) were collected and tested again by removing the blood clot from the tail tip at the indicated time points after the first test. If no more blood could be collected from the mouse tail tip, it was cut for another 1 mm for blood sampling.

A control group was included in the present study, for which the P7-8 neonatal mice were removed from the parents’ cages and left in the experiment room for the same periods of time as the anesthetized group. Additional control mouse pups were placed in the chamber filled with a mixture of O_2_ and N_2_ (50%/50%) at a flow rate of 0.9–1 L/min without anesthetics. This group was included for assessing whether breathing with an O_2_ concentration higher than that in the air has any impact on the blood glucose level.

For injection anesthesia, 150 mg/kg propofol or 15 μl/g of 2.5% avertin was administered through intraperitoneal injection. Under these doses, propofol led to anesthesia of mouse pups for 6–7 hrs, and avertin led to full anesthesia for 3–4 hrs, followed by 1–2 hrs of sedative status.

Unless specified, at least 9 mouse pups were included in each group of the study. To eliminate any potential bias caused by litter variations, a similar number of mouse pups from each litter was assigned to each group, and each group included pups from several litters.

For collecting blood samples for insulin ELISA assays, the mouse pups were sacrificed at the indicated time points by decapitation, and approximately 250 μl blood was collected into 1.5-ml centrifuge tubes through the neck. After allowing the blood samples to stay at room temperature for ~5 min, they were centrifuged at 2,000 *g* at 4°C for 20 min, and the sera were collected and stored at -80°C for ELISA at a later date.

The adult mice (male, 2–3 months old) used for this study were first habituated to handling by the investigator for 14 days prior to the experiment. Food, but not water, was removed from the cages for 4 hrs before anesthesia with 2.5% sevoflurane, as described for neonatal mice, for up to 6 hrs. Control adult mice were kept in the same experiment room with water, but no food, available for the same periods as when the anesthetized mice were not fed (during 6-hr anesthesia and 1-hr recovery). Tail blood glucose was measured at 0, 2, 4, 6, 7, 8 and 10 hrs after the start of anesthesia.

### Intranasal saline administration and intraperitoneal glucose injection

Some mouse pups were administered a total 7.0 μl saline through intranasal delivery. Briefly, the P7-8 mouse pups were held in a supine position in hand, and 0.7 μl saline was delivered into the left side nare using a 10 μl Eppendorf pipette. The pups were given ~20 seconds to allow the fluid inhalation before repeating the administration for up to 10 times. The mouse pups were then anesthetized 30 min later with sevoflurane for 6.0 hrs, as described above. Some of these mouse pups were given a single dose of glucose (0.5 mg/g body weight) through intraperitoneal injection 4 hrs after the start of anesthesia.

### Serum insulin measurement

Serum insulin level was assayed by using the ultra-sensitive mouse insulin ELISA (Crystal Chem, Catalog #90080; Downers Grove, IL) according to the manufacturer’s instructions.

### Statistical analysis

All data were analyzed by repeated measures ANOVA followed by Bonferroni post hoc tests using Graphpad. All data are presented as means ± SEM, and *p*<0.05 was considered statistically significant.

## Results

### Anesthesia with sevoflurane induces severe hypoglycemia in neonatal mice

**Fig 1:**
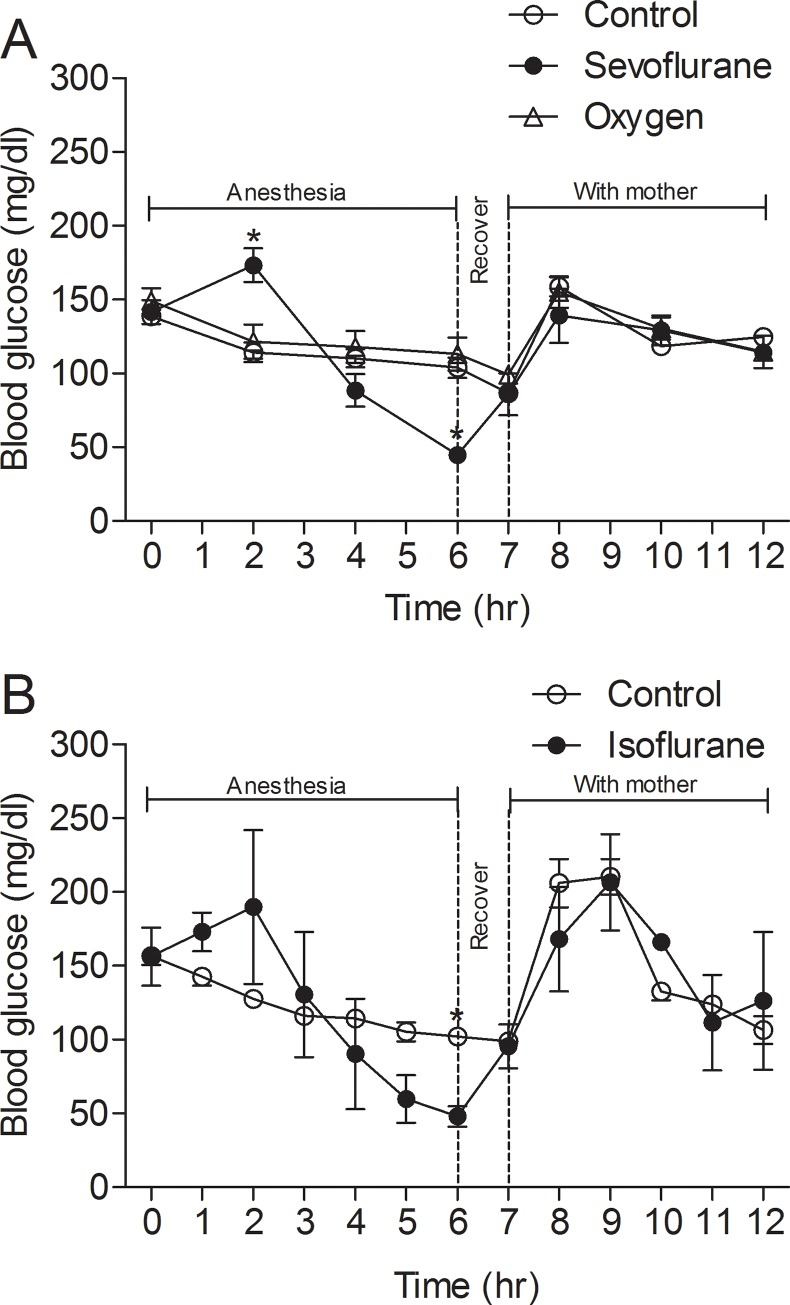
Dynamic change of blood glucose level of neonatal nice before, during, and after anesthesia with sevoflurane or isoflurane. (**A**) P7-8 mice were kept in an anesthesia chamber filled with a mixed gas of 50% O_2_ and 50% N_2_ containing either 0% (oxygen group, n = 6) or 2.5% sevoflurane (Sevo group, n = 7) at a flow rate of 0.9–1 L/min. After anesthesia for 6 hrs, the sevoflurane supply was terminated, and the mouse pups were kept in the chamber for an additional 1 hr for recovery, followed by return to their mothers in their home cages. For a control group (n = 7), the neonatal mice were separated from their mothers and kept in the experiment room for 7 hrs before being returned to their mothers in their home cages. Tail blood glucose level was measured every 1 or 2 hrs. (**B**) Sevoflurane was replaced with 1.5% isoflurane (n = 4) under the conditions identical to (A). *p < 0.05 vs. control and oxygen group.

We monitored the tail blood glucose level of P7-8 neonatal mice before, during, and after anesthesia with sevoflurane by using a glucometer. We found a slight increase (~25%) of blood glucose level (from 141.50 ± 8.10 mg/dl to 173.20 ± 11.45 mg/dl, *p* = 0.003) after anesthesia of mouse pups for 2 hrs (Fig 1A), which is likely a response to stress induced by anesthesia, as reported previously [3, 4]. However, after this time point, the blood glucose level decreased dramatically and reached 44.60 ± 5.18 mg/dl (nearly 70% reduction from the level prior to anesthesia, *p* = 0.0001) with continuous anesthesia for 6 hrs. The blood glucose level started to increase immediately after the termination of sevoflurane and reached the same level as the control mouse pups, although the mouse pups were not fed during the 1-hr recovery phase. This result further supports that the hypoglycemia of the sevoflurane group is caused by the anesthetic rather than by fasting. The blood glucose level of the control group declined only slightly (from 138.54 ± 3.10 mg/dl to 103.77 ± 6.96 mg/dl, *p* = 0.0001) during the same 6-hr period, which represented a normal decline due to lack of feeding (Fig 1A). The blood glucose level of mouse pups in both sevoflurane and control groups returned to the normal level within 1 hr post feeding by their mothers. These results indicate a remarkable and progressive decline of blood glucose level in mouse pups, after an initial slight increase, during anesthesia with sevoflurane.

It has been reported that oxygen content in the breathing air may affect blood glucose level [6]. Because 50% O_2_ was used to carry sevoflurane in the present study, we wondered whether the difference in blood glucose levels between the anesthetized group and the control group, which were left in the experiment room with 21% O_2_ in the air, was due to the different O_2_ content they breathed. Thus, we included another group, in which mouse pups were put in the same chamber filled with a mixture of O_2_ and N_2_ (50%/50%) but no sevoflurane for the same period as for the anesthetized group. We found no significant difference in blood glucose levels between this oxygen group and the control group (Fig 1A). This result further supports that the changes in blood glucose level of the sevoflurane group is caused by the anesthetic.

### Anesthesia-induced hypoglycemia in neonatal mice is unique to inhalational anesthetics

**Fig 2:**
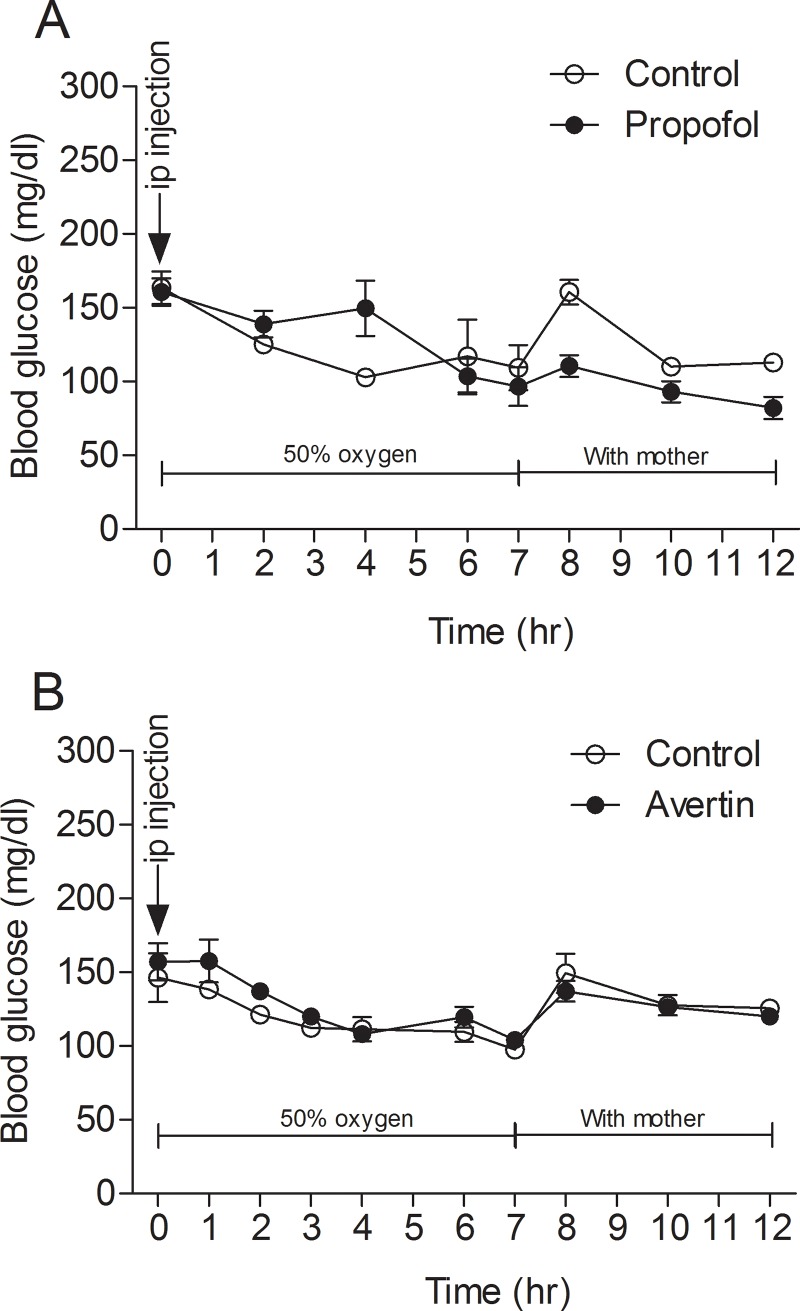
Blood glucose level of neonatal mice before, during, and after anesthesia with propofol or avertin. P7-8 mice were treated with intraperitoneal injection of propofol (150 mg/kg body weight) (**A**) or avertin (375 mg/kg body weight) (**B**) and immediately placed in an anesthesia chamber filled with 50% O_2_ and 50% N_2_ at a flow rate of 0.9–1.0 L/min for up to 7 hrs before they were returned to their mothers. Tail blood glucose level was measured every 1–2 hrs. For the control group, the mouse pups were separated from their mothers and placed in the experiment room for 7 hrs before they were returned to their parents in their home cages. *p < 0.05 vs control.

To learn whether the hypoglycemia induced by sevoflurane is a common feature to general anesthesia in neonatal mice, we investigated the blood glucose levels of mouse pups after anesthesia with different anesthetics, including one other volatile anesthetic (isoflurane) and two different injection anesthetics (propofol and avertin). We found that after an initial slight elevation to 189.75 ± 52.13 mg/dl (*p* = 0.278), isoflurane caused the same changes in blood glucose level (i.e., marked decline to 48.00 ± 6.95 mg/dl, *p* = 0.0006) (Fig 1B) as sevoflurane did. However, no such decrease in blood glucose level was seen after the mouse pups were anesthetized with propofol or avertin as compared with the control groups (*p* = 0.320 and 0.338, respectively) (Fig 2A and 2B). These findings indicate that the hypoglycemia-induced by anesthesia in neonatal mice is not a common feature of general anesthesia. Instead, it is unique to volatile anesthetics and does not occur if anesthesia is induced by injectable anesthetics such as propofol and avertin.

### Sevoflurane induces hyperglycemia instead in adult mice

**Fig 3:**
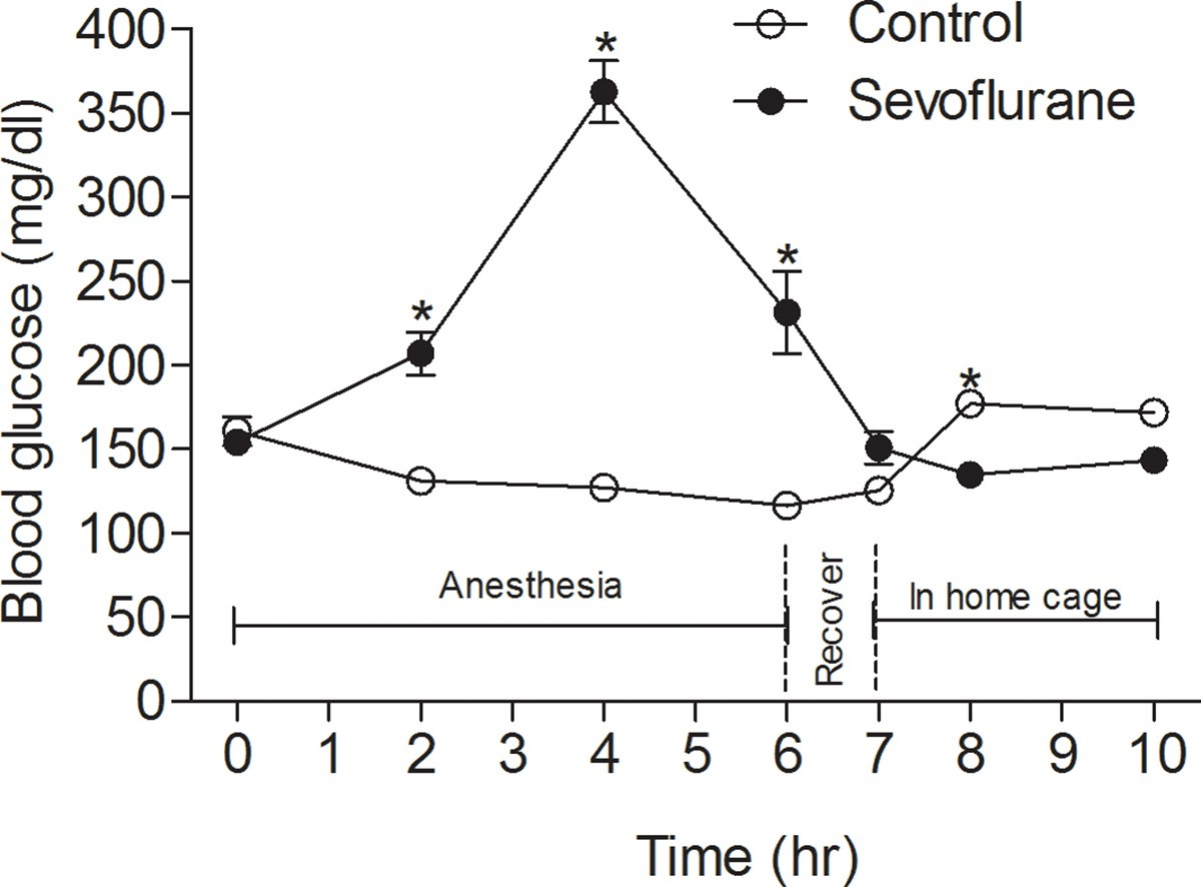
Dynamic changes in blood glucose level in adult mice before, during and after anesthesia with sevoflurane. Young adult male mice (2–3 months old) were kept in an anesthesia chamber filled with a mixed gas of 40% O_2_ and 60% N_2_ containing 2.5% sevoflurane (n = 14) at a flow rate of 0.9–1 L/min for up to 6 hrs. The sevoflurane supply was then terminated, and the mice were kept in the chamber for an additional 1 hr for recovery, followed by their return to their home cages with food and water. The control mice (n = 12) were also moved into the experiment room with free access to water, but not food, for 7 hrs before they were returned to their home cages. Tail blood glucose level was measured every 1–2 hrs. *p < 0.05 vs control.

To investigate whether sevoflurane-induced hypoglycemia is unique to neonatal mice or it occurs in adult mice as well, we anesthetized adult mice with sevoflurane and monitored the blood glucose levels. The control mice were left in the same experiment room with free access to water but not to food, because the anesthetized group did not feed during anesthesia and recovery time. We found that contrary to the hypoglycemia observed in neonatal mice, sevoflurane induced a reversible elevation of blood glucose level, from 154.14 ± 6.90 mg/dl to 362.93 ± 18.57 mg/dl (~2.3-fold increase, *p* < 0.0001) at 4 hrs of continuous anesthesia (Fig 3). The blood glucose level started to decline after anesthesia for 4 hrs and reached the normal level 1 hr after the termination of sevoflurane. As expected, the blood glucose level of the control mice declined only slightly (from 160.75 ± 8.58 mg/dl to 116.50 ± 6.58 mg/dl, *p* = 0.0005) during the 7-hr fasting period and returned to the normal level after re-feeding. These results indicate that the sevoflurane-induced severe hypoglycemia is unique to neonatal mice.

### Serum insulin level changes along with the blood glucose level during anesthesia in neonatal mice

**Fig 4:**
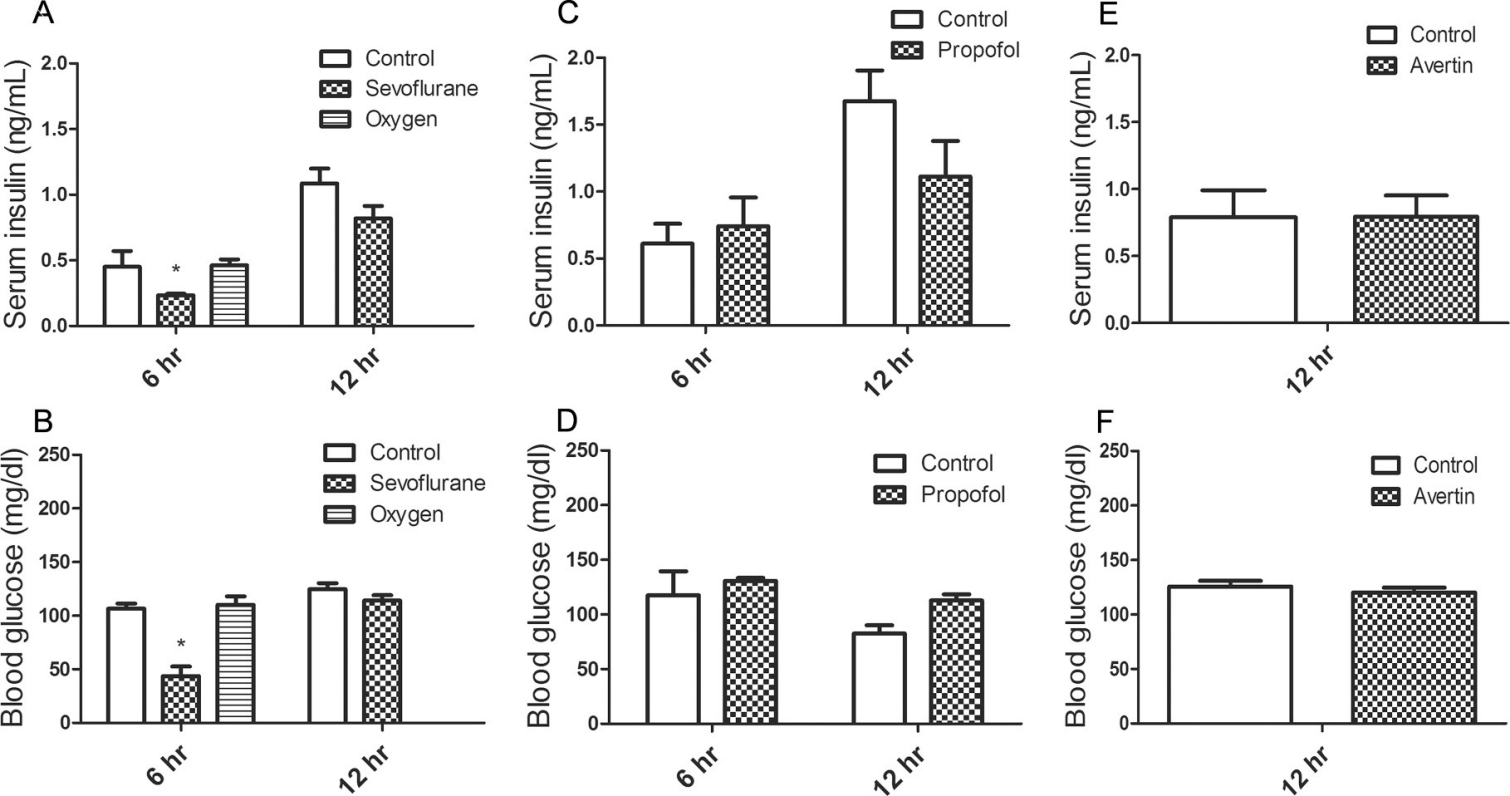
Levels of serum insulin and blood glucose in neonatal mice post-anesthesia and recovery. (**A,B**) P7-8 mouse pups were anesthetized with sevoflurane for 6 hrs, followed by 1-hr recovery and return to their parents. The serum insulin (**A**) and blood glucose (**B**) levels were measured after continuous anesthesia for 6 hrs and, using separate mice, at 6 hrs after the termination of anesthesia (12-hr point). (**C,D**) Serum insulin (**C**) and blood glucose (**D**) levels of neonatal mice measured at 6 and 12 hrs post-propofol injection. (**E,F**) Serum insulin (**E**) and blood glucose (**F**) levels of mouse pups measured at 12 hrs post-avertin injection. N = 6/group; *p < 0.05 vs control.

Insulin is the most important regulator of blood glucose level. Thus, we measured serum insulin level in mouse pups at the end of continuous anesthesia with sevoflurane for 6 hrs and again at 6 hrs after the termination of anesthesia (12-hr time point shown in Fig 4). We found a dramatic reduction (~50%) in serum insulin level in the anesthetized (for 6 hrs) mouse pups as compared to the control pups (from 0.45 ± 0.12 ng/ml to 0.23 ± 0.01 ng/ml, *p* = 0.005) (Fig 4A). The extent of this reduction of serum insulin was similar to the reduction of blood glucose in the anesthetized pups (from 106.67 ± 4.67 mg/dl to 43.67 ± 8.95 mg/dl, *p* = 0.002) (Fig 4B). No significant changes (*p* = 0.942 and 0.738, respectively) were found in the levels of either serum insulin or blood glucose in the 50% oxygen control group (Fig 4A and 4B). These results suggest that the reduction of serum insulin in the mouse pups after anesthesia for 6 hrs may be a response to the sevoflurane-induced reduction of blood glucose. After the mouse pups were returned to their mothers and fed, their blood glucose levels returned to normal levels, and their serum insulin level post-anesthesia was not significantly different from that of the control group (0.82 ± 0.09 ng/ml vs. 1.09 ± 0.11 ng/ml, *p* = 0.082) (Fig 4A). However, the insulin level in both groups at 6 hrs post-anesthesia (the 12-hr time point) was higher than that at the end of anesthesia (the 6-hr time point) (Fig 4A), which probably represents elevated insulin secretion post feeding.

There were no significant differences (0.74 ± 0.22 ng/ml vs. 0.61 ± 0.15 ng/ml, *p* = 0.651) in the serum insulin level at 6 hrs post-injection between the propofol group and the control group (Fig 4C), which is consistent with the similar blood glucose level in the two groups (130.67 ± 2.85 mg/dl vs. 111.67 ± 21.80 mg/dl, *p* = 0.586) (Fig 4D). The serum insulin level of these mouse pups at 12 hrs post-propofol injection (1.11 ± 0.26 ng/ml) was found to be higher than that at 6 hrs post propofol injection (0.74 ± 0.22 ng/ml, *p* = 0.394) (Fig 4C), representing the elevation of blood insulin in response to feeding, which started at 7 hrs post-propofol injection. No change in either serum insulin level or blood glucose level was observed in the mouse pups 12 hrs post-avertin as compared to the control-injected pups (*p* = 0.964) (Fig 4E and 4F). These results together further support that the reduction of serum insulin in the mouse pups after anesthesia with sevoflurane for 6 hrs is probably a response to the sevoflurane-induced reduction of blood glucose.

### Glucose administration reduces the mortality during sevoflurane-induced anesthesia

**Fig 5:**
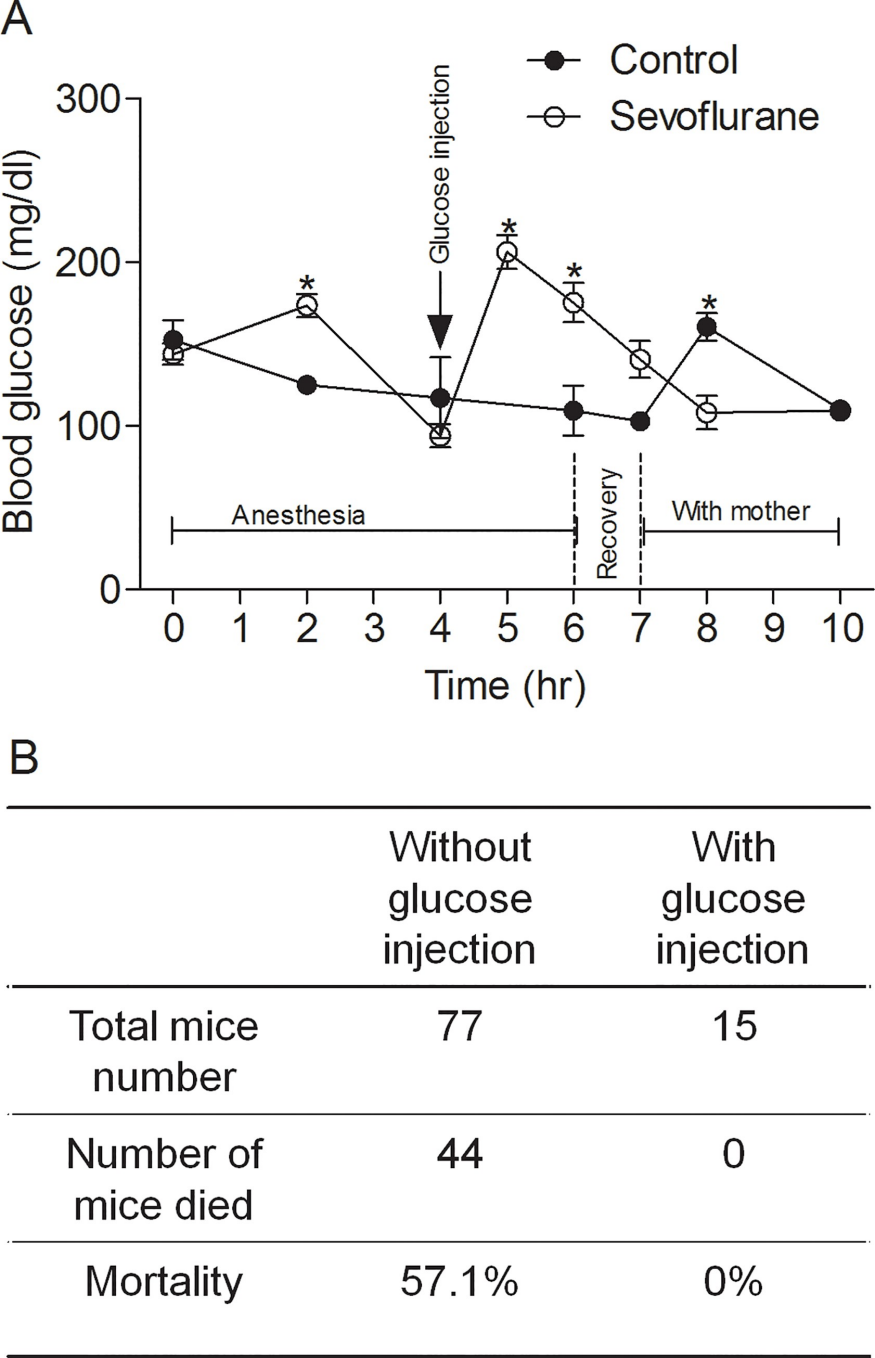
Mortality of mouse pups during anesthesia with sevoflurane. P7-8 mouse pups (n = 77) were administrated with intranasal saline, followed by anesthesia with sevoflurane for 6 hrs. After termination of sevoflurane for 1 hr for recovery, the mouse pups were returned to their parents in their home cages. Another group (n = 15) received a single dose of glucose (0.5 mg/g body weight) through intraperitoneal injection 4 hrs after the start of anesthesia. (**A**) Blood glucose level of the mouse pups during and post anesthesia. *p < 0.05 vs. control group. (**B**) The number of mouse pups died during anesthesia.

Anesthesia of P7-8 mouse pups with sevoflurane for 6 hrs did not lead to death under our conditions. However, high mortality was observed if mouse pups received intranasal administration of insulin or saline 30 min before anesthesia when we worked on another project aimed to investigate the preventative role of intranasal administration of insulin against anesthesia-induced neurotoxicity in neonatal mice. In light of our findings that sevoflurane induced severe hypoglycemia as described above, we wondered whether the hypoglycemia contributes to the mortality and whether correction of the hypoglycemia can prevent the mortality. Thus, we administered glucose through intraperitoneal injection after the mouse pups received intranasal administration of saline and then anesthesia with sevoflurane for 4 hrs, the time point when hypoglycemia occurred, to prevent the progression of hypoglycemia to the severe level. Blood glucose measurement indicated that the glucose administration retained the blood glucose level within the range of 100–200 mg/dl during the remaining 2-hr anesthesia and the recovery phase (Fig 5A). We found that the prevention of severe hypoglycemia through glucose administration eliminated the mortality of the mouse pups during anesthesia with sevoflurane (Fig 5B), whereas 57.1% of mouse pups without glucose administration died during anesthesia under the same conditions. These results suggest that severe hypoglycemia contributes to the high mortality of mouse pups with both intranasal drug administration and sevoflurane-induced anesthesia.

## Discussion

Sevoflurane is the most commonly used inhalational anesthetic in clinical practice, including for children. However, the impact of the anesthesia on the metabolism in children, especially in infants, has not been well investigated. In the present study, we found that sevoflurane and isoflurane, which are two commonly used inhalational anesthetics, induced severe hypoglycemia during anesthesia in neonatal mice but not in adult mice. This phenomenon appears to be unique to the halogenated ether family of anesthetics because hypoglycemia was not observed in neonatal mice during anesthesia with propofol or avertin. These results point to the potential risk of dangerous hypoglycemia occurring during anesthesia of infants with the halogenated ether family of anesthetics.

Previous studies have reported a temporary increase, rather than decrease, of blood glucose during anesthesia of adult rodents with sevoflurane or isoflurane, which is consistent with our data with adult mice. Zuurbier et al. found that the inhalational anesthetics sevoflurane and isoflurane per se, without surgical insult, induce hyperglycemia in rats partly through impaired glucose-induced insulin release [7]. Marked hyperglycemia has also been observed after sigmoid colostomy under sevoflurane anesthesia [8]. Prolonged anesthesia with sevoflurane causes glucose intolerance due to diminished insulin output in response to blood glucose elevation during surgery [9]. However, no changes in blood glucose were observed during and after anesthesia in 55-day-old lambs with sevoflurane [10]. A previous overlooked study that was not focused on blood glucose level reported that anesthesia of P7 neonatal mice with isoflurane for six hours resulted in reversible hypoglycemia [5]. This report is consistent with our results that suggest that the effect of inhalational anesthetics on blood glucose level is different and opposite between infants and adults.

At present, the exact molecular mechanisms of general anesthesia are incompletely understood. We currently do not know why the inhalational anesthetics sevoflurane and isoflurane, but not the injectable anesthetics propofol and avertin, induce severe hypoglycemia in neonatal mice. Because the inhalational anesthetics may act on cell membranes, in addition to GABA receptors [11–15], the sevoflurane/isoflurane-induced hypoglycemia might result from disruption of plasma membrane integrity rather than specific membrane receptors or targets. Another explanation of the mechanism of general anesthesia is the lateral membrane pressure profile theory hypothesizing that general anesthetics may act through changes of the lateral pressure profile [16]. We thus speculate that the inhalational anesthetics may disrupt the cell plasma membrane and/or lateral pressure profile, which might in turn alter glucose transporters, so that they transport more glucose from the blood stream into the cell, leading to hypoglycemia. It was reported that sevoflurane increases glucose uptake in skeletal muscle cells, which is associated with tyrosine kinase, protein kinase C, and intracellular calcium [17]. Whether glucose transport across plasma membrane is affected by lateral membrane pressure profile has not been reported and requires investigation in the future. The reversible nature of hypoglycemia we observed is consistent with the fact that the disruption of neuronal cell membrane is reversible. This hypothesis is consistent with our observation that this phenomenon occurred only in neonatal mice but not in adult mice, since the plasma membrane in the newborn is very different from than that of adults [18].

In contrast to the hypoglycemia observed in neonatal mice, we found that anesthesia of adult mice led to marked hyperglycemia that peaked at four hours after continuous anesthesia. This marked increase in blood glucose level likely results from both anesthesia-induced stress and resistance to insulin [19, 20]. Impaired glucose-induced insulin release and hyperglycemia were found in rats under anesthesia with sevoflurane or isoflurane [8]. Increased glucose production and decreased glucose utilization were also reported during anesthesia with isoflurane anesthesia [21]. Human studies also showed impaired insulin secretion and glucose utilization and increased blood glucose level in adults during anesthesia with sevoflurane or isoflurane [19, 20, 22].

Insulin is the most important hormone regulating blood glucose level. We thus measured blood insulin level of neonatal mice during and post anesthesia. We found a marked decline in blood insulin level when the blood glucose level was low in neonatal mice during anesthesia with sevoflurane or isoflurane. Because the decrease in insulin level was found to correspond to that of blood glucose level, this alteration of blood insulin is possibly due to decreased insulin secretion in response to anesthesia-induced hypoglycemia in neonatal mice. However, a possible change in insulin sensitivity during anesthesia of neonatal mice cannot be excluded.

We do not currently know whether the hypoglycemia induced by inhalational anesthesia has any long-term impact. Increased apoptotic cellular degeneration and mild behavioral abnormalities occurring later in life after neonatal mice are exposed to anesthesia with sevoflurane have been reported [23], but whether these alterations resulted from or are related to hypoglycemia remains elusive. Hypoglycemia induced by inhalational anesthesia in neonatal mice appears to make them more vulnerable to other insults, since high mortality of neonatal mice during anesthesia with sevoflurane after intranasal administration of saline can be eliminated if the severe hypoglycemia was prevented by a single dose of glucose administration after anesthesia for 4 hrs (Fig 5). These results suggest that severe hypoglycemia contributes to the high mortality of mouse pups that received both intranasal administration and sevoflurane-induced anesthesia. Although saline is benign and not toxic, intranasal administration itself appears to temporarily affect breathing of neonatal mice, which alone did not lead to mortality without anesthesia. However, the combination of partial interruption of breathing and anesthesia-induced severe hypoglycemia could lead to high mortality of neonatal mice.

In conclusion, we found severe hypoglycemia during general anesthesia of neonatal mice with inhalational anesthetics but not with injectable anesthetics. Our findings would warrant close monitoring blood glucose level and maintaining it within the normal range in infants receiving inhalational anesthesia. Sevoflurane and isoflurane are commonly used for pediatric anesthesia, but blood glucose level is not routinely monitored during inhalational anesthesia of infants. If the severe hypoglycemia that we found in neonatal mice also occur in infants during anesthesia with inhalational anesthetics, a change in clinical practice that will require close monitoring and prevention of hypoglycemia must be considered.
